# *FMR1* Disorders: Basics of Biology and Therapeutics in Development

**DOI:** 10.3390/cells13242100

**Published:** 2024-12-18

**Authors:** Drew A. Gillett, Helene Tigro, Yuan Wang, Zucai Suo

**Affiliations:** Department of Biomedical Sciences, College of Medicine, Florida State University, Tallahassee, FL 32306, USA

**Keywords:** fragile X syndrome, fragile X messenger ribonucleoprotein 1, *FMR1*, fragile X messenger ribonucleoprotein, FMRP, neuronal development, FXS therapeutics, extracellular vesicles, exosomes

## Abstract

Fragile X Syndrome (FXS) presents with a constellation of phenotypes, including trouble regulating emotion and aggressive behaviors, disordered sleep, intellectual impairments, and atypical physical development. Genetic study of the X chromosome revealed that substantial repeat expansion of the 5′ end of the gene fragile X messenger ribonucleoprotein 1 (*FMR1*) promoted DNA methylation and, consequently, silenced expression of *FMR1*. Further analysis proved that shorter repeat expansions in *FMR1* also manifested in disease at later stages in life. Treatment and therapy options do exist, but they only manage symptoms. Up to now, no cure for *FMR1* disorders exists. In this review, we aim to provide an overview of *FMR1* biology and the latest research focused on developing therapeutic interventions that can potentially prevent and/or reverse FXS.

## 1. Introduction

Originally described in 1943 by James Purdon Martin and Julia Bell, Fragile X Syndrome (FXS) is a congenital developmental disorder and is the most common cause of inherited intellectual disability [1,2]. Research completed in 1991 demonstrated that a portion of the X chromosome had an unstable region of trinucleotide repeats in the X chromosome [3,4]. Further investigation revealed that trinucleotide repeat expansions in the promoter of the gene fragile X messenger ribonucleoprotein 1 (*FMR1*) enhances methylation of the expanded promoter region and, consequently, silences expression of *FMR1* [1,5]. The size of the repeat expansion influences the consequence on human health, where larger repeat expansions have an impact earlier in the lifespan, like FXS in childhood, and smaller expansions, called premutation alleles, have a delayed onset, like Fragile X-Associated Primary Ovarian Insufficiency (FXPOI) or Fragile X-Associated Tremor/Ataxia Syndrome (FXTAS) in young and late adulthood, respectively [6,7]. Symptoms of FXS can include intellectual impairments, behavioral problems, and atypical physical development, among others [1,2]. The loss of the fragile X messenger ribonucleoprotein (FMRP), encoded by the *FMR1* gene, has drastic effects on human development, particularly in both the central and peripheral nervous systems (CNS and PNS, respectively) [1,8,9]. Shorter repeat expansions associated with FXPOI and FXTAS do not influence FMRP expression but, through mechanisms that are still being elucidated, influence human health over the lifespan [1,5].

The known structure and biology of FMRP and *FMR1* disorders have been well reviewed by other groups [2,10,11,12,13,14]. However, the understanding of the field is incomplete, and, accordingly, current pharmacological and therapy options only manage symptoms [1,2]. This review will discuss the relevant biology of FXS and other *FMR1* disorders and also examine an opportunity for the development of a novel therapeutic strategy using extracellular vesicles (EVs).

## 2. *FMR1* Gene

Located at q27.3 on the X chromosome, *FMR1* is approximately 40 kb [4,5]. Encoded across 17 exons, *FMR1* is notable for the instability of the untranslated regions (UTRs), undergoing both trinucleotide repeat expansions and contractions in the 5′ and 3′ UTRs [15]. Approximately 5–44 trinucleotide repeats are considered normal, while 45–54 repeats and 55–200 repeats are, respectively, labeled intermediate and premutation alleles [1,2,6]. Intermediate alleles have no reported effect on human health. Premutation alleles do not impact CNS development but may manifest in mid to late adulthood as FXPOI or FXTAS, respectively [6,7]. In contrast, FXS manifests in childhood and is the result of a full mutation allele, an FMR1 allele with over 200 trinucleotide repeats [5].

*FMR1* alleles are inherited through the X chromosome. Assuming only one *FMR1* allele is expanded, parents with two X chromosomes (XX parents) have a 50% chance of passing down an expanded FMR1 allele to their child. Parents with only one X chromosome (XY parents) have a 100% chance of passing down the expanded allele on their X chromosome to their XX child but a 0% chance of passing down the allele to their XY child, who only receives an X chromosome from their XX parent. Multiple factors influence the stability of the gene during transmission, including the sex of the transmitting parent, the size of the repeat, and how the repeats are structured [15,16]. For normal and intermediate alleles, the *FMR1* gene transmitted from an XY parent was more likely to be unstable (be expanded or contracted) than an *FMR1* gene transmitted from an XX parent [15]. *FMR1* premutation alleles from XY parents are more likely to be maintained as premutation alleles. Premutation alleles from XX parents, however, often expand into a full mutation over time. Another factor that influences the transmission of *FMR1* is the number and location of an interrupting AGG trinucleotide repeat. The majority of the repeats are CGG trinucleotide repeats, but AGG trinucleotide repeat interruptions influence the stability of the gene [15,16]. Specifically, an AGG repeat interruption at the 11th triplicate position was found to be more stably transmitted than an AGG repeat interruption at the 10th triplicate position [15].

## 3. FMRP Protein—Structure, Function, and Expression

**Figure 1:**
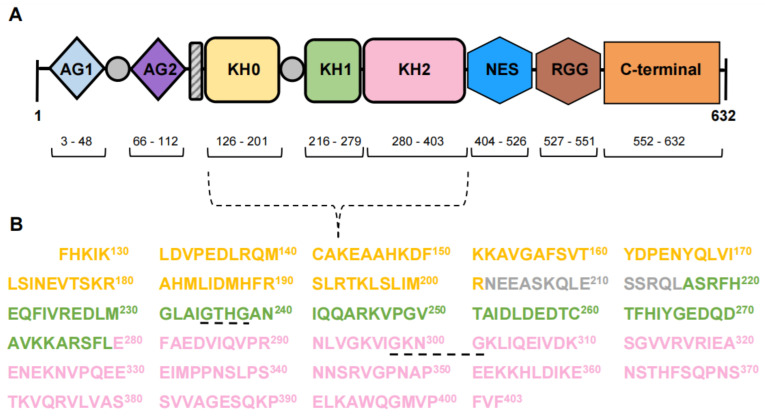
FMRP domain organization and amino acid sequences. (**A**) Diagram of the domains of FMRP, including Agenet domains (AG1 and AG2), several unstructured regions (shown in gray), a nuclear localization signal (NLS, shown in striped gray), KH domains (KH0, KH1, and KH2), a nuclear export sequence (NES), a RGG box, and the C-terminal domain. (**B**) Amino acid sequences of the KH0, an unstructured region; KH1, and KH2 are in yellow, grey, green, and pink, respectively. GXXG motifs (G^235^THG^238^ in KH1, G^298^KNG^301^ in KH2) are underlined.

**Figure 2:**
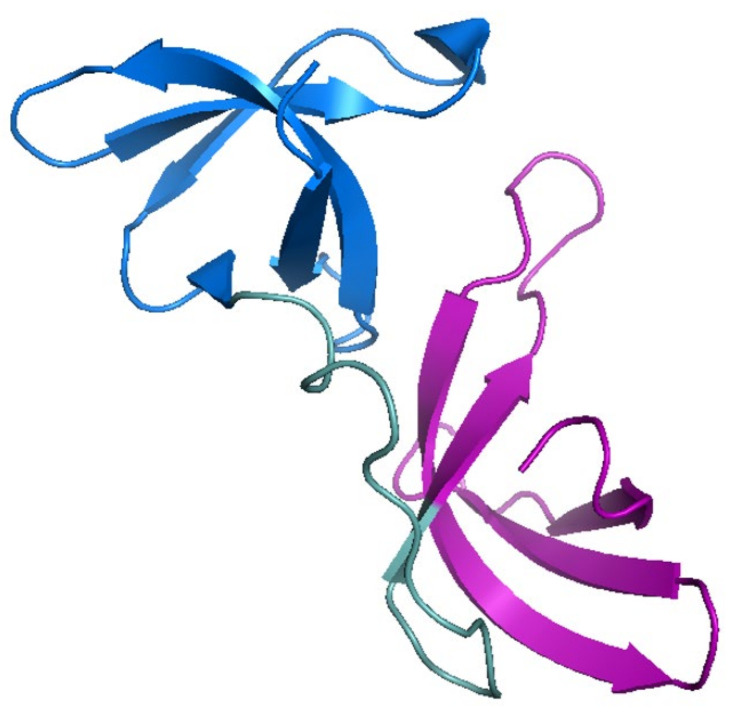
Crystal structure of the Agenet domains of human FMRP (PDB code: 4QW2). The structures of AG1, AG2, and an unstructured region between them are shown in blue, purple, and green respectively.

**Figure 3:**
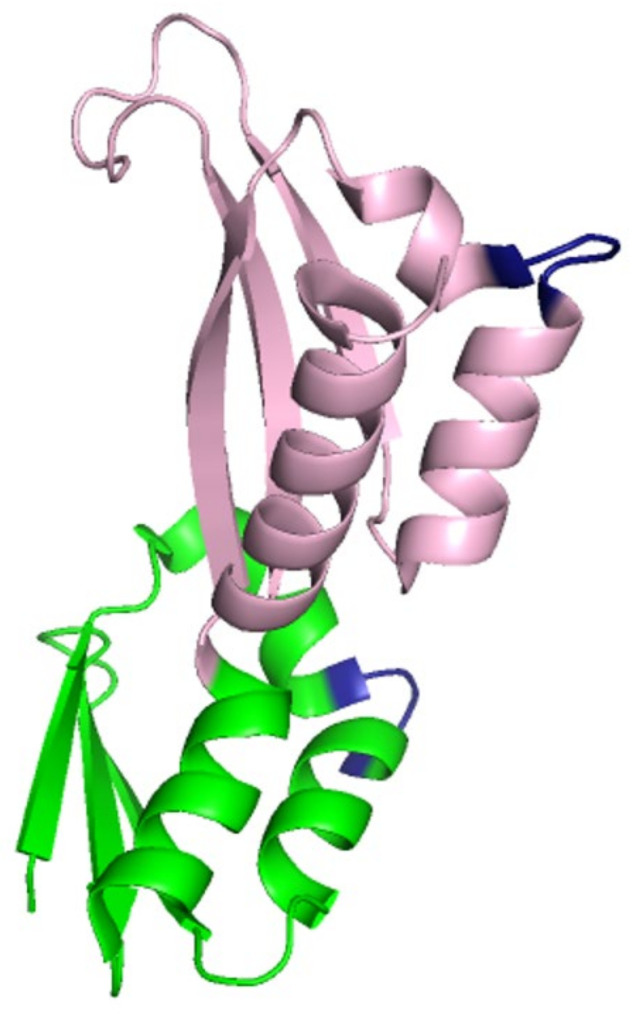
Crystal Structure of the KH1-KH2 domains of human FMRP (PDB code: 2QND). The structures of KH1 and KH2 are shown in green and pink, respectively. The GXXG motifs are shown in dark blue.

Full-length FMRP is a 632-amino acid residue protein [10,13]. The structure and organization of distinct domains of FMRP (Figure 1A) allow it to interact with other proteins and various forms of RNA, including mRNAs, microRNAs, and small non-coding RNAs. The N-terminal 200 amino acid residues form the motifs that are primarily used in protein–protein interactions (Figure 1A). Two Agenet-like domains (also called Tudor domains), AG1 and AG2, are each made up of a four-stranded anti-parallel β-sheet with a fifth strand closing around the curved β-sheet [10,17] (Figure 2). The two Agenet-like domains are similar in structure, but the second domain has been demonstrated to be more flexible than the first and is considered to be the surface that allows protein–protein interactions [10,18]. The Nuclear Localization Signal (NLS), is another motif that permits protein–protein interactions (Figure 1A). In this case, the NLS sequence interacts with importin and Ran, which are required for entry into the nucleus [19]. The last motif in the N-terminal 200 amino acid residues is the first of three hnRNP K-homology (KH) RNA binding domains, KH0 (Figure 1) [18,20]. This domain has a distinct primary sequence, not only from the other two KH domains in FMRP (Figure 1B), but also from KH domains on other RNA-binding proteins [18]. The typical conserved structure of a KH domain is composed of a β-sheet made of three antiparallel β-strands adjacent to three α-helices and also contains a β1α1α2β2 fold with a GXXG motif on the loop that connects α1 to α2 [20] (Figure 3). KH0 does not contain the GXXG motif (Figure 1B), indicating that another mechanism of RNA recognition is present [18]. In addition, AG1 and KH0 are thought to work together to interact with RNA while AG2 interacts with methylated lysine and arginine residues in interacting protein partners [17,18].

Two additional KH domains, KH1 and KH2, are located in the central region of FMRP (Figure 1A) [10]. They have a high degree of sequence similarity (Figure 1B) and are connected to KH0 with a loop (Figure 1B) [20]. The RNA-binding capabilities of the central region are critical for human development as a point mutation in these motifs is linked to FXS [21]. A Nuclear Export Signal (NES) is the last motif in the central region (Figure 1A) and coordinates the removal of FMRP from the nucleus.

The C-terminal region of FMRP (Figure 1A) is intrinsically disordered with the exception of an arginine–glycine–glycine (RGG) box that allows FMRP to bind to G quartets, structural RNA formations that are rich in guanine nucleotides, and many microRNAs [22,23,24].

During early fetal development, FMRP is highly expressed throughout the body, but as the fetus matures, FMRP expression decreases to almost negligible levels except in the brain and the reproductive system [25]. After birth, FMRP continues to play a critical role in CNS development. FMRP binds to mRNA transcripts, regulating the transcription, processing, and translation of the bound mRNAs [26,27,28]. Recent work identified a subset of mRNA transcripts bound by FMRP by immunoprecipitating FMRP and sequencing the bound mRNA transcripts [29]. Biological processing analysis using gene ontology (GO) terms identified several pathways associated with cell morphogenesis and neuronal development, which supports the conclusions of an earlier analysis of FMRP-bound mRNA transcripts [29,30].

Insufficient regulation by FMRP causes an increase in neuronal translation of a subset of its mRNA targets but simultaneously downregulates the translation of another subset of mRNA targets [26,27,28]. The RGG box of FMRP is the most well studied RNA-binding motif, where methylation of the arginine modulates the binding of the G quartets’ RNA structure(s) [22]. The KH2 domain has also been shown to bind mRNA-associated polyribosomes, forming a kissing stem-loop that binds a hairpin of an mRNA with non-specific interactions [31]. The KH1 domain is thought to have a similar function, as an FXS-linked missense mutation, G266E, blocks mRNA binding, likely through charge–charge repulsion, and prevents association with polyribosomes [30].

In addition to regulating protein translation, FMRP also regulates the stability, localization, and maturation of its mRNA transcripts by binding the post-transcriptional modification, N6-methyladenosine [32,33,34]. Furthermore, multiple models demonstrate that FMRP interacts with the protein named adenosine deaminase acting on RNA (ADAR) [35,36,37]. The activity of ADAR converts adenosine to inosine, which is read as guanosine, and this swap can cause amino acid substitutions to occur, which in turn promotes protein diversity [38]. The full implications of the FMRP–ADAR interaction and the extent that it influences the proteome is still being explored.

FMRP interacts with other proteins, primarily a subset of channel proteins or proteins that also bind and regulate RNAs [39,40]. Notable among the published interaction partners is cytoplasmic interaction FMRP protein 1 (CYFIP1). The complex of CYFIP1, FMRP, and eukaryotic translation initiation factor 4E (eIF4E) is critical to regulating translation, and the loss of either FMRP or CYFIP1 causes FXS [41]. FMRP also interacts with the network of proteins that form ribonucleoprotein (RNP) particles and stress granules (SG) [42]. RNP particles transport paused mRNA transcripts in order to regulate the spatial and temporal translation of the bound mRNA at synapses that are significant distances away from the nucleus. Stress granules are a dynamic response to the loss of homeostasis, and their successful formation and decomposition are critical to cell survival.

The coordination of these dynamic structures relies, in part, on the post-translational modification(s) (PTMs) of FMRP. Phosphorylated FMRP associates with ribosome-bound mRNA and represses translation, while dephosphorylation disassociates FMRP from ribosome-bound mRNA and permits translation [14,43,44]. Similarly, ubiquitination, and the subsequent degradation, of FMRP is necessary for the translation of neuronal proteins related to long-term depression (LTD) [14,45]. SUMOylation of FMRP also accomplishes a similar goal, disassociating FMRP from bound mRNA and allowing translation of the bound mRNA [14,46]. Methylation, however, has a less overt effect of FMRP activity, and the methylation of specific arginine residues may prompt FMRP to bind different subsets of both mRNA transcripts and proteins, further specifying the function(s) of FMRP [14,47,48]. The number and functional overlap of these PTMs are critical to regulating the activity of different isoforms of FMRP that may have different PTM sites. In the adult mouse brain, for example, 8 isoforms of FMRP are reported but only 2 isoforms have the Ser-499 residue thought to be the canonical phosphorylation site for FMRP regulation [49].

## 4. *FMR1* and Human Health

Beyond a specific threshold, increased trinucleotide repeats in the promoter region of *FMR1* have consequences on human health and development. FXS is an inherited intellectual disability that results in altered behavior, impaired cognition, and atypical physical development [1,2]. Prenatal diagnostic testing can be performed in utero by amniocentesis at 16 to 20 weeks or by chorionic villus sampling (CVS) at 10 to 13 weeks. After birth, early signs in very young individuals with FXS are delayed milestone achievements: learning to sit up, walk, and talk at older ages than their peers [1,2]. FXS can be diagnosed as early as 12 months of age using these behavioral phenotypes. As individuals with FXS age, they continue to exhibit behavior that is distinct from their peers [1,2]. Individuals with FXS may experience increased anxiety, sensory hypersensitivity, and seizures, in some cases [1,2].

Socially, individuals with FXS may struggle to interact normally with their peers due to speech problems, repeated movements (such as hand flapping or other stimming behaviors), aggressive behavior, abrupt speech or actions, and difficulty maintaining eye contact [1,2]. Individuals with FXS also display cognitive disabilities that can vary from mild learning disabilities or attention difficulties to more severe intellectual impairments [1,2]. Physical abnormalities associated with FXS, a large head and ears with a narrow face and prominent forehead and flat feet, are typically absent at a young age but begin to develop during puberty and become more pronounced with age [1,2].

As an X-linked syndrome, FXS is more common in individuals with only one X chromosome, and thus only one copy of *FMR1*, than those with two [1,2]. The estimated prevalence of FXS in people with one X chromosome is 1 in 4000 to 1 in 7000, while the prevalence of FXS in people with two X chromosomes is estimated as 1 in 6000 to 1 in 11,000 [1,2]. However, even when present, the symptoms of FXS are less severe, and may even appear absent, in people with two X chromosomes [1,2]. The factors that modulate the penetrance of the impacted allele in people with two X chromosomes are unknown.

Even in the absence of FXS, expansions in the *FMR1* gene can still result in disease. Premutation alleles, *FMR1* alleles that have anywhere from 55 to 200 trinucleotide repeats, are associated with FXPOI and FXTAS [50]. FXPOI is defined as hypergonadotropic hypogonadism before the age of 40. While FXPOI only occurs in 1% of the general population, 20% of XX individuals with a premutation allele will develop FXPOI [50]. Genetic testing can confirm the presence or absence of an *FMR1* premutation allele, but little is known about the mechanism(s) behind FXPOI. Hormone-replacement therapy (HRT) designed for menopause is currently the predominant treatment to encourage regular menses and prevent osteoporosis [50,51].

FXTAS is a late-onset movement disorder classified by intention tremor and progressive cerebellar ataxia [50]. Typical age of onset is between 60–65 years old, but symptom severity and progression are highly heterogeneous based on factors like sex and repeat length [50]. Consistent with other X-linked diseases, FXTAS is both more frequent and more severe in XY individuals than XX individuals, and longer repeat length is associated with increased symptom severity [50]. Tremor is typically the first reported symptom, but as the disease progresses, cognitive impairments and ataxia also develop [12,50]. A definite diagnosis can be determined using both radiological signs and clinical signs or only using neuropathologic staining for intranuclear ubiquitin-positive inclusions with low or absent signal for tau or alpha-synuclein [12,50]. Further investigation revealed that these inclusions are enriched with both *FMR1* mRNA and other proteasome proteins [11,12]. These inclusions have also been found in the peripheral nervous system and other organs like the testes, heart, and kidney and may contribute to other symptoms documented in FXTAS patients such as cardiac arrythmias, swallowing problems, erectile dysfunction, and/or constipation [11,12,52]. A variety of mechanisms of cell toxicity have been proposed, but none have identified specific therapeutic targets [11,53,54]. Presently, no disease-modifying treatments are available for FXTAS or any *FMR1* disorders, and care is focused on managing symptoms. Further research into *FMR1* biology and how repeat length influences the likelihood of developing disease is sorely needed.

## 5. *FMR1* Therapeutics in Clinical Trials and Development

Currently, treatment for individuals with FXS only manage symptoms. Early intervention with special education and combinations of speech, behavioral, and occupational therapy are foundational to better outcomes later in life [1,2]. Pharmacological treatment can also be used to supplement treatment of FXS symptoms, primarily anxiety, sleep, attention, and behavioral symptoms [1,2]. FXS has little impact on the life span, so individuals with FXS can live long, relatively healthy lives with appropriate support from caregivers, therapists, and physicians [1,2].

The history of clinical trials for FXS shows a pattern of using existing drugs that modulate neurotransmitter activity, like Memantine, Gaboxadol, and Sertraline, in an attempt to regulate symptoms of anxiety, attention deficiency, and social withdrawal in individuals with FXS [55,56,57]. Other trials make use of CNS-acting drugs like psilocybin and cannabidiol to achieve similar effects [58,59].

Connecta Therapeutics has implemented AI to design a first-in-class small molecule drug for treating FXS [60]. CTH120, labeled as a “neuroplasticity modulator”, is in an active Phase 1 clinical trial to determine not only the therapeutic effects on individuals with FXS but also the safety and tolerability of a therapeutic dose [60]. The precise mechanism(s) involved in the effects of CTH120 have not been publicly disseminated at this time.

Zatolmilast (BPN14770), a phosphodiesterase-4D (PDE4D) inhibitor developed by Tetra Therapeutics, is being investigated as a potential treatment for individuals with FXS. In Phase 2 clinical trials, BPN14770 demonstrated a favorable safety profile, with no significant differences in adverse events between the treatment and placebo groups [61]. Moreover, the drug showed significant cognitive improvement in domains related to language with corresponding improvement in caregiver scales rating language and daily functioning [61]. Encouraged by these findings, Shionogi & Co. (Osaka, Japan) has advanced BPN14770 to Phase 3 clinical trials.

Zygel (ZYN002), a cannabidiol (CBD) transdermal gel, has been developed by Zynerba Pharmaceuticals to potentially treat children and adolescents with FXS. A Phase 2 clinical trial showed good tolerability for ZYN002, with promising results in improving social avoidance behaviors specifically in a subgroup of patients with ≥90% *FMR1* gene methylation, who typically experience more severe FXS symptoms [62]. Based on these positive outcomes, ZYN002 has advanced to Phase 3 clinical trials to further explore its potential as a treatment for FXS.

Treatment with metformin, a drug used in type 2 diabetics, was found to be efficacious in an *FMR1* KO mouse model [63,64,65], which sparked multiple studies of metformin treatment in individuals with FXS. Results varied, but, overall, both adult and juvenile individuals with FXS showed improvement with metformin treatment, with younger individuals with FXS showing greater improvement in language and behavior with metformin treatment [64]. The mechanism behind this effect is not completely clear, but metformin is believed to cool the increased translation and intracellular signaling cascades (e.g., ERK, mTOR) that are elevated in the CNS of individuals with FXS [65].

Repurposing old drugs to manage symptoms is a worthy goal, but the heterogeneity of *FMR1* disorders, particularly in individuals with two X chromosomes, highlights the need for a therapeutic approach that addresses the common cause, the defective *FMR1* allele. Given the genetic cause of FXS, gene therapy is a natural avenue of treatment, but this approach has produced mixed results. Supplementation of a functional *FMR1* gene using the intravenous injection of a recombinant Adeno-Associated Virus (rAAV) in adult mice was shown to be successful in both accessing the CNS and generating FMRP [66]. However, restoration of FMRP to neurons in 6-week-old *Fmr1* KO mice only had modest effects in anxiety-like behaviors and learning and memory paradigms relative to empty-vector treated mice [66]. In contrast, the intracerebroventricular injection of the rAAV construct in *Fmr1* KO pups was able to partially rescue the behavioral phenotypes that were observed in *Fmr1* KO pups treated with an empty vector AAV [67]. The disparity between these results likely stems from the stage of development when the exogenous *FMR1* gene was introduced as well as the neuronal populations that were successfully transduced by the rAAV construct given the different routes of administration. Both of these points highlight the possibility of a specific therapeutic window for *FMR1* replacement strategies, which will be important considerations in adapting therapeutics to human use. Other studies have identified another variable with *FMR1* gene therapy: *FMR1* is naturally spliced into many isoforms in both mice and humans [49,68,69,70]. Replacing the heterogeneous, and likely dynamic, mixture of *FMR1* isoforms with only a single isoform is likely to result in only a partial rescue, which is consistent with gene therapy studies in a mouse model of FXS [67,71,72,73]. Further research regarding gene therapy of specific *FMR1* isoforms is ongoing but has produced mixed results thus far [71,74].

An alternative approach to FMRP replacement does not use exogenous *FMR1*, but instead rescues the endogenous *FMR1* mRNA. *FMR1-217* is an isoform of *FMR1* that is generated by dysfunctional splicing of *FMR1* and is present in a subpopulation of individuals with FXS [75]. The effects of the retained intron are not fully understood, but the transcript may be rescued to produce a viable FMRP with anti-sense oligonucleotides (ASOs) [75]. The ASOs block the retention of the intron during mRNA processing, and the resulting mRNA can be translated to produce FMRP [75]. This presents a novel mechanism of intervention for FXS that may produce viable therapeutic alternatives to traditional gene therapy; however, this approach will not be suitable for every FXS patient as only a subpopulation has the *FMR1-217* isoform required for the ASO-mediated rescue.

## 6. Opportunities for Innovation

Therapeutics based on the genetics of FXS may produce viable treatment strategies, but other avenues to amend the loss of FMRP and/or address the other mechanisms that arise from the expansion of the *FMR1* primer region must be considered. Enzyme replacement therapy has been demonstrated as a viable therapeutic approach for Lysosomal Storage Diseases (LSDs) [76]. Recently, this simple principle of directly supplementing the necessary enzyme(s) has been successfully expanded to a protein replacement therapy in a mouse model of Frontotemporal Dementia and Neuronal Ceroid Lipofuscinosis (NCL-11) [77]. In the context of FMRP, this approach runs into a similar problem as gene therapy, where the isoform(s) supplied may not be sufficient to rescue or modify every phenotype associated with FXS. To address this challenge, a potential solution lies in precision medicine, where patients could be treated based on the specific FMRP isoforms they are deficient in. A more favorable substitute for FMRP protein may involve the direct delivery of *FMR1* mRNA. By using viral vectors or lipid nanoparticles (LNPs), *FMR1* mRNA can be delivered to target cells where it can be spliced into the required isoforms to meet the dynamic needs of the cells. For the first method, viral vectors, such as lentiviruses and adeno-associated viruses, can be engineered to target specific cell types or brain regions and are highly efficient at delivering FMR1 mRNA into cells, enabling long-term expression of the FMRP protein. This method has limitations, including potential immunogenicity and the risk of insertional mutagenesis, both associated with viral vectors. For the second method, lipid nanoparticle-based *FMR1* mRNA delivery to the brain offers several advantages, including targeted delivery to specific cell types, such as neurons, and rapid, transient expression of the FMRP protein. Notably, nanoparticle drug delivery systems (DDS) have gained popularity in recent years following the successful use of LNPs to deliver the COVID-19 vaccine [78]. However, this method also has limitations, including the potential to trigger an immune response, off-target delivery to unintended brain cells, and the inherent instability of the delivered *FMR1* mRNA, which can lead to degradation by cellular enzymes.

In contrast to supplementing FMRP protein in individuals with FXS, inhibiting the expression of *FMR1* using an ASO or microRNA may be beneficial in preventing the formation and/or progression of the *FMR1* mRNA inclusions in FXTAS patients [79,80,81]. Additionally, the same approach can be used to target the repeat-associated non-ATG (RAN) translation that has been proposed to be a toxic mechanism in FXTAS [80,82].

However, a notable challenge in all of the abovementioned therapeutic approaches is the successful delivery of intact and functional therapeutics to the appropriate cell populations without incurring significant loss to circulating peripheral myeloid cells and/or extracellular proteases or nucleases. To achieve a similar goal, naturally occurring EVs are well-suited for therapeutic use. EVs are defined as particles that are released from cells, are delimited by a lipid bilayer, and cannot replicate on their own” [83].

**Figure 4:**
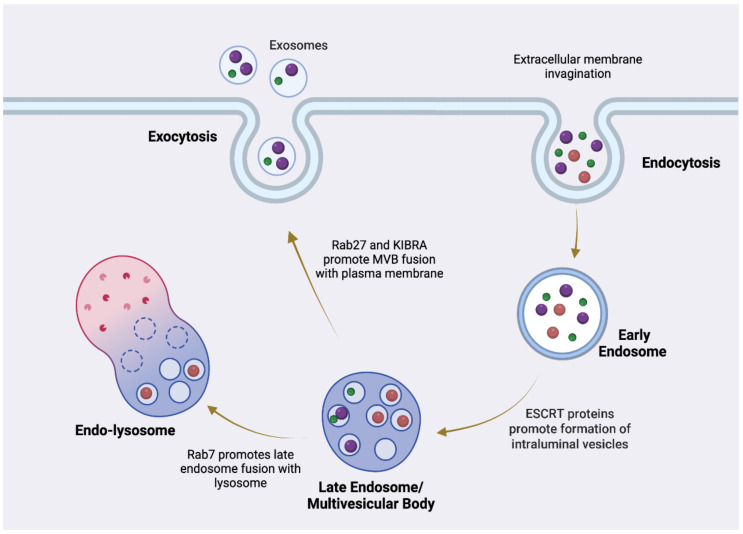
Diagram of exosome formation and release in the endosomal system. Generated using Biorender.Com.

**Figure 5:**
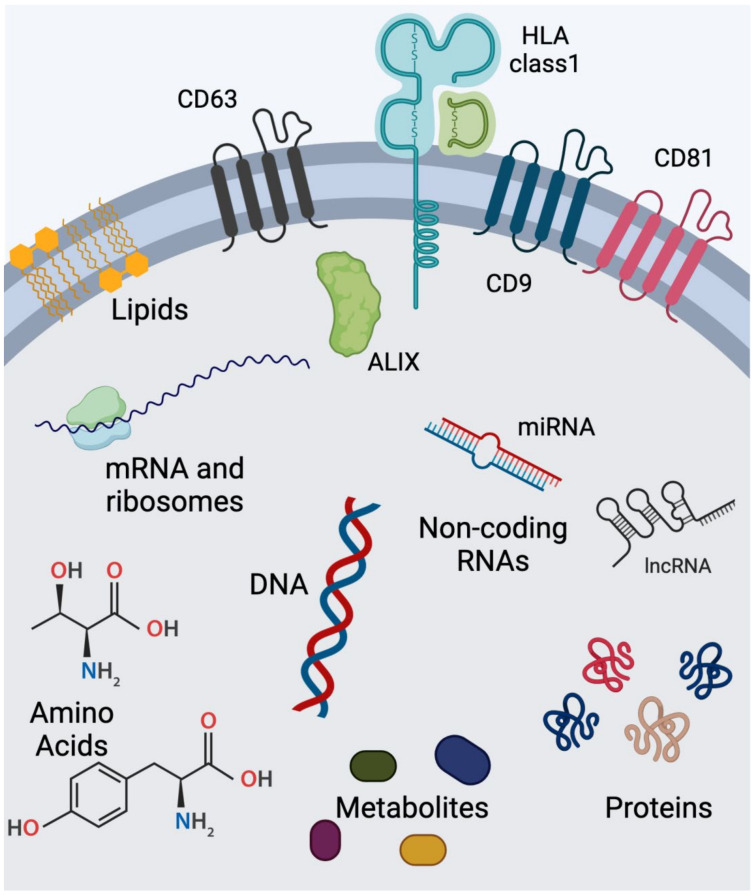
Diagram of a representative endogenous exosome and its membrane markers and cargos. Generated using Biorender.com.

Exosomes, the smallest EVs, begin with the invagination of the plasma membrane and the formation of an early endosome (Figure 4) [84]. As endosomes mature, cargos are sorted between other subtypes of endosomes with specialized endpoints in the endocytic pathway, and some endosomes will develop intralumenal vesicles (ILVs) and mature into a late endosome/multivesicular body (MVB) [85,86]. Endosomal cargos may be sent out of the cell entirely, trafficked intracellularly to the Golgi network in order to be reused, or, if the cargos are marked for destruction (ubiquitinated), trafficked to the lysosomal system for degradation [85]. For most cells, Endosomal Sorting Complexes Required for Transportation 0 (ESCRT-0) protein, located on the endosome membrane, binds to the cargo(s) and phosphatidylinositol 3-phosphate (PI3P) to initiate the process [84,87]. Working together, ESCRT-I and ESCRT-II recruit ESCRT-III to deform the endosome membrane and generate intralumenal vesicles (ILVs) [84,87,88]. ALG-2-interacting protein X (ALIX) also participates in ILV formation by interacting with ESCRT-III, which may be the predominant mechanism in generating ILVs that will be released as exosomes given that ALIX is a canonical EV marker [84,87,88]. Throughout this sorting process, Rab GTPases assist in marking the endosome/MVB and recruiting effector trafficking proteins [86,89,90]. In particular, Rab27 is predominantly associated with MVBs fated for the secretion of EVs, and recent evidence has elucidated that KIBRA, a scaffold protein, binds to Rab27 and blocks the ubiquitination and subsequent degradation of Rab27, promoting exosome secretion [91] (Figure 4). Exosomes are typically 30–100 nanometers in diameter and contain a variety of cargo molecules, including proteins, lipids, metabolites, small molecules, and nucleic acids (Figure 5).

EVs are released by cells in both health and disease but can be generated for therapeutic use and offer a number of benefits in the delivery of therapeutics [92,93,94]. Early nanoparticle DDS experienced issues regarding water-solubility and immunogenicity, but exosomes are readily compatible with human cells and offer a minimal risk of rejection from the patient [95,96]. Furthermore, the encapsulation of the therapeutic(s), such as *FMR1* mRNA, inside the exosomes may minimize first-pass metabolic activity and permit the delivery of the therapeutic cargo(s) to specific body compartments or tissues, e.g., the brain, without degradation from extracellular enzymes or peripheral immune cell activity [95,96,97,98]. This modular approach to exosome-mediated therapy permits greater flexibility into both the therapeutic cargo(s) delivered and the cell population(s) targeted.

Finally, EVs have been documented to bypass the Blood–Brain Barrier (BBB), a significant challenge that has doomed many promising CNS-therapeutics [99,100,101,102]. In a mouse model of Alzheimer’s Disease (AD), intranasal delivery of EVs isolated from mesenchymal stem cells ameliorated the AD-like phenotype, and the inflammation and memory dysfunction elicited by a status epilepticus model were reduced with EV treatment [103,104]. Similar beneficial therapeutic effects have been recorded in both stroke and traumatic brain injury (TBI) models [105,106,107]. The intranasal administration route appears to be optimal for CNS-targeted therapeutics, and the conjugation of an additional targeting moiety on the surface of the EVs can further improve the specificity of the EV-mediated delivery, simultaneously improving the precision of delivering a therapeutic dose and preventing undesirable off-target effects and even toxicity [108,109].

We believe that utilizing EVs to target the delivery of therapeutics—protein or enzyme replacement, small-molecule drugs, mRNA, microRNA, and ASOs—is a significant opportunity to innovate in developing and optimizing non-viral treatments for diseases currently considered incurable, e.g., FXS.

## Data Availability

No original data was generated for this review.
